# Mitogen- and Stress-Activated Protein Kinase 1 Mediates Alcohol-Upregulated Transcription of Brf1 and tRNA Genes to Cause Phenotypic Alteration

**DOI:** 10.1155/2020/2067959

**Published:** 2020-06-27

**Authors:** Mingen Lin, Chenghao Huang, Wenfeng Ren, Jun Chen, Ningshao Xia, Shuping Zhong

**Affiliations:** Keck School of Medicine, University of Southern California, Los Angeles, CA, USA

## Abstract

Upregulation of Brf1 (TFIIB-related factor 1) and Pol III gene (RNA polymerase III-dependent gene, such as tRNAs and 5S rRNA) activities is associated with cell transformation and tumor development. Alcohol intake causes liver injury, such as steatosis, inflammation, fibrosis, and cirrhosis, which enhances the risk of HCC development. However, the mechanism of alcohol-promoted HCC remains to be explored. We have designed the complementary research system, which is composed of cell lines, an animal model, human samples, and experiments *in vivo* and *in vitro*, to carry out this project by using molecular biological, biochemical, and cellular biological approaches. It is a unique system to explore the mechanism of alcohol-associated HCC. Our results indicate that alcohol upregulates Brf1 and Pol III gene (tRNAs and 5S rRNA) transcription in primary mouse hepatocytes, immortalized mouse hepatocyte-AML-12 cells, and engineered human HepG2-ADH cells. Alcohol activates MSK1 to upregulate expression of Brf1 and Pol III genes, while inhibiting MSK1 reduces transcription of Brf1 and Pol III genes in alcohol-treated cells. The inhibitor of MSK1, SB-747651A, decreases the rates of cell proliferation and colony formation. Alcohol feeding promotes liver tumor development of the mouse. These results, for the first time, show the identification of the alcohol-response promoter fragment of the Pol III gene key transcription factor, Brf1. Our studies demonstrate that Brf1 expression is elevated in HCC tumor tissues of mice and humans. Alcohol increases cellular levels of Brf1, resulting in enhancement of Pol III gene transcription in hepatocytes through MSK1. Our mechanism analysis has demonstrated that alcohol-caused high-response fragment of the Brf1 promoter is at p-382/+109bp. The MSK1 inhibitor SB-747651A is an effective reagent to repress alcohol-induced cell proliferation and colony formation, which is a potential pharmaceutical agent. Developing this inhibitor as a therapeutic approach will benefit alcohol-associated HCC patients.

## 1. Introduction

Alcohol is a very common beverage. Alcohol drinks are consumed day by day for many persons. Studies have demonstrated that alcohol consumption induces liver injury, such as steatosis, inflammation, fibrosis and cirrhosis, and elevates hepatocellular carcinoma (HCC) risk [1]. Alcohol combines with hepatitis virus C or B, carcinogens, and others to facilitate HCC development [2–6]. Although the relationship of alcohol consumption with HCC has been widely studied, the mechanism of alcohol-associated HCC remains to be explored. Early studies have indicated that chronic alcohol intake produces acetaldehyde and induces cytochrome P450 2E1 (CYP2E1). Alcohol dehydrogenase (ADH) catalyzes ethanol to acetaldehyde by dehydrogenation, while acetaldehyde has direct roles of mutagen and carcinogen [6]. CYP2E1 plays important roles in the release of reactive oxygen species (ROS) and procarcinogens converting to carcinogens. Alcohol intake increases the cellular level of ROS, resulting in cell stress which causes liver injury and alcoholic liver disease (ALD). In spite of the exact mechanism of alcohol-promoted HCC still being unclear, alcohol intake induces liver carcinogenesis by different mechanisms: mutagenesis by acetaldehyde, oxidative damage, and reducing folic acid intake and usage. Our early study has demonstrated that alcohol affects TFIIB-related factor 1 (Brf1) expression and RNA polymerase III-dependent gene (Pol III gene) transcription [7], which is responsible for biosynthesis of proteins and is associated with transformation and tumorigenesis.

Pol III gene products, such as tRNAs and 5S rRNA, are increased in transformed cells and tumor tissues; it implies that these products play an important role in tumorigenesis [7–11]. Brf1 is a component of transcription factor III B complex (TFIIIB) of Pol III gene transcript machinery. Brf1 specifically and directly regulates tRNA and 5S rRNA transcription. Repressing Brf1 expression decreases transcription of Pol III genes, leading to inhibition of cell transformation and xerograph growth [12–15]. Our studies have revealed that Brf1 is highly expressed in human tumor tissues of HCC cases [16]. The cases of HCC with high Brf1 expression display a short survival time [16]. Transcription of Brf1 and Pol III genes is additionally enhanced in the cases of HCC with alcohol intake, compared to those of nonalcohol consumption. This suggests that alcohol is associated with HCC development. Our further studies have indicated that alcohol enhances the levels of Brf1 and Pol III gene activities of HepG2-ADH cells and that increases in Brf1 and Pol III gene expression promote liver tumor formation in HCV nonstructural 5A protein (NS5A) transgenic mice fed with alcohol [7]. This implies that Brf1 may be a new target of alcohol and it may play an important role in alcohol-associated HCC development.

Studies have demonstrated that alcohol activates MAP kinases, which modulate Brf1 and Pol III genes [7, 17, 18]. Mitogen- and stress-activated protein kinase 1 (MSK1) is a serine/threonine protein kinase in cell nuclei. It is a downstream component of the MAP kinase pathway. MSK1 modulates gene expression and cell transformation [19–23]. MSK knockout mouse has no significant health problems. Knockout MSK represses skin cancer development of mice [24, 25]. Our previous studies and others have demonstrated that MSK1 mediates histone H3 phosphorylation (H3ph) [26, 27]. We have reported that H3ph modulates the transcription of Brf1 and Pol III genes [14, 15]. Inhibiting H3ph decreases the expression of Brf1 and Pol III genes, leading to repression of cell proliferation and transformation [14, 15]. Together, these studies suggest that inhibiting Brf1 expression should suppress HCC development. Up to now, there have been no reports on the roles of MSK1 in alcohol-induced Brf1 and Pol III gene expression.

In the present study, our results indicate that alcohol markedly induces MSK1 activation. Inhibiting MSK1 reduces alcohol-increased cellular levels of Brf1 and Pol III gene products. Alcohol increases the rates of cell proliferation and colony formation. In contrast, blocking the MSK1 pathway represses cell growth and colony formation. Mechanism analysis indicates that alcohol enhances Brf1 promoter activity through MSK1. Further analysis reveals that alcohol-caused high-response part of the Brf1 promoter is at the p-382/+109 region. These studies, for the first time, report the identification of alcohol-induced response region in the Brf1 promoter. These outcomes from this paper provide a possibility to develop a specific and efficient inhibitor to repress Brf1 expression as a novel therapeutic approach for HCC patients.

## 2. Materials and Methods

### 2.1. Cell Lines, Reagents, Antibodies, and Animals

Primary mouse hepatocytes (PMHs) were isolated by the Cell Biology Core Laboratory of Research Center for Liver Diseases of University of Southern California (P30 DK48522). Immortalized mouse hepatocytes, AML-12 line, and human HepG2 cell line were from ATCC (Manassas, Virginia, USA). Human HepG2-ADH and HepG2-vector cells were from Dr. D.L. Clemens (University of Nebraska) [28]. Ethanol was ordered from Sigma-Aldrich. Cell culture media (DMEM), G418, Lipofectin reagent, Lipofectamine 2000, TRIzol reagent, and Opti-MEM were from Thermo Fisher (San Diego, CA, USA). The antibody of actin was obtained from Santa Cruz Biotech (Santa Cruz, CA, USA). Brf1 antibody was from Bethyl Laboratories Inc. (Montgomery, TX, USA). Horseradish peroxidase-conjugated secondary antibodies of mouse and rabbit were from Cell Signaling Technology (Danvers, MA). MTT assay kit was from Boster Bio (Pleasanton, CA). The sequences of human Brf1 siRNA and mismatch RNA (MM siRNA) are described as previously [14, 15]. HCV NS5A transgenic mice were originally from Ratna Ray (Saint Louis University). The C57BL/6 strain of NS5A transgenic mice was fed with a Lieber-DeCarli diet containing 3.5% ethanol or isocaloric dextrin for long-term alcohol feeding [7]. The experiments performed with age- and sex-matched mice from same littermates were described as before [7, 29]. Conduction of the experiment was approved by the Institutional Animal Care and Use Committee of University of Southern California.

### 2.2. Real-Time qPCR and Transfection Assays

Total RNAs were extracted from PMHs, AML-12 cells, and HepG2-vector and HepG2-ADH cells with TRIzol reagent (Life Technology). The precursor of tRNA^Leu^ and 5S rRNA transcripts and Brf1 mRNA were determined by real-time qPCR (RT-qPCR) [30]. For transient transfection assays, HepG2-ADH cells and AML-12 cells were transfected with plasmid DNAs or siRNAs [7]. A none-serum medium was added into each dish with Lipofectin-DNA or Lipofectamine 2000-siRNA, and cells were further grown in 37°C with 5% CO_2_ for 4 h. And then, the cells were grown in 10% FBS/DMEM for 48 h before harvesting. Cell lysates were extracted from the cells to determine protein concentrations by the Bradford method using a Fluostar Omega spectrometer (Cell Biology Core Laboratory of Research Center for Liver Diseases of USC, P30 DK48522).

### 2.3. Cell Proliferation and Colony Formation

Approximately 2 × 10^3^ HepG2-ADH cells were seeded in 6-well plates in triplicate. Cells were treated with MSK1 inhibitor and ethanol. The cells were determined for viability and counted each day for 5-6 days using a Coulter counter or MTT assay (Boster Biotech) [30, 31]. HepG2-ADH cells (1 × 10^4^ cells/well in 6-well plate) were suspended in 0.35% (*w*/*v*) agar in 10% FBS/DMEM with or without the inhibitor and 50 mM ethanol. The bottom layer was in the media with 0.5% (*w*/*v*) agar and ethanol. The cells were cultured in complete media with inhibitor plus ethanol twice weekly. Colony numbers were counted around 2 weeks after plating [15, 31].

### 2.4. Brf1-Luciferase Reporter Assays

HepG2-ADH cells were transfected with 0.2 *μ*g plasmids of Brf1-Luc (p-182/+109bp, p-382/+109bp, or p-760/+109bp) as described [7, 8]. 2 ml of none-serum medium was filled to each 6 cm dish with Lipofectamine 2000-plasmid complexes, and cells were cultured at 37°C with 5% CO_2_ for 4 h. The medium was removed, and fresh 10% FBS/DMEM [7] were added into the dishes. Cells were incubated at 37°C with 5% CO_2_ for 48 h before harvesting. The cells were starved in none-serum DMEM for 4 hours with MSK1 inhibitor, SB-747651A. The cells were further treated with 50 mM ethanol for another 2 hours. The pellets of cells were collected with a Promega reporter lysis buffer. The luciferase activity in the resultant lysates was measured using a luminometer and the Promega Luciferase Assay System as described (Promega). The resultant lysate protein concentrations were determined using the Bradford method. Luciferase activities of these lysates were normalized to protein amounts of each sample as described [7, 8]. The fold change was calculated by measuring the level of luciferase activity in the absence of alcohol; its value was set at 1 for each independent experiment. Means ± SE represent at least three independent experiments.

### 2.5. Immunoblot Analysis

Cells were grown to 85% confluency in 10% FBS/DMEM. The cells were cultured in none-serum DMEM for 4 h with SB-747651A. And then, the cells were treated with ethanol (50 mM) for 2 hours to isolate cell lysates. Lysates (50 *μ*g of protein) were added to SDS-PAGE, and immunoblot analysis was performed as previously described [15, 16]. The transferred membranes with the proteins were probed with the first antibodies as indicated. Bound primary antibody was visualized using horseradish peroxidase-conjugated secondary antibody (Cell Signaling Tech.) and enhanced chemiluminescence reagents (Santa Cruz Biotech).

### 2.6. Immunohistochemistry

Immunohistochemical staining was carried out on formalin-fixed, paraffin-embedded sections (4 *μ*m thick) which were deparaffinized in xylene and rehydrated in decreasing concentrations of ethanol and rinsed in phosphate-buffered saline and then retrieval antigen with microwave treatment in 10 mM citrate buffer (pH 6.0). Immunohistochemistry staining was carried out using the EnVision™ Kit (DAKO, Hamburg, Denmark) following the manufacturer's instruction. The endogenous peroxidase activity was quenched by 3% hydrogen peroxide for 15 minutes. The sections were incubated with primary antibody, Brf1 (1 : 200) rabbit antibody, overnight at 4°C [32]. Then, the tissue sections were sequentially incubated with ready to use HRP-immunoglobulin (EnVision™) for 30 min and were developed with 3,3′-diaminobenzidine (DAB) as a chromogen substrate. The nuclei were counterstained with Meyer's hematoxylin [16, 32].

## 3. Results

### 3.1. Alcohol-Caused Upregulation of Brf1 and Pol III Gene Transcription

**Figure 1:**
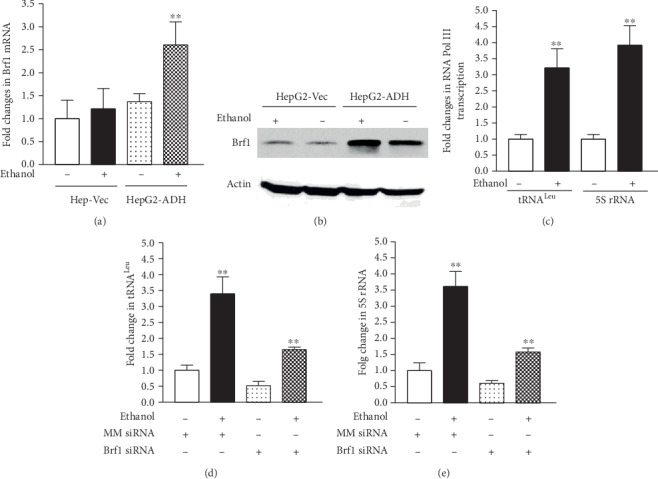
Alcohol increases Brf1 expression and Pol III gene transcription. (a, b) HepG2-ADH and HepG2-vector cells were grown in 10%FBS/DMEM to 85% confluency and starved in FBS-free DMEM for 4 hours and then treated with 50 mM ethanol for another 2 hours. Total RNA and protein were extracted from the cells. Brf1 mRNA (a) and protein (b) were determined by RT-qPCR and immunoblot assays. A representative immunoblot is shown in (b). (c) The cellular levels of pre-tRNA^Leu^ (c, left panel) and 5S rRNA (c, right panel). (d, e) HepG2-ADH cells were transfected with mismatch siRNA (MM siRNA as a control) or Brf1 siRNA and then treated with 50 mM ethanol. RT-qPCR were used to determine the levels of pre-tRNA^Leu^ (d) and 5S rRNA (e). The fold changes are calculated by normalizing to the amount of GAPDH mRNA. The bars represent mean ± SE of at least three independent determinations. ^∗∗^*p* < 0.01.

As HepG2 cells lost ADH or express trace amounts of ADH, we used the engineered HepG2 cells which were restored with ADH expression constructs [28]. The engineered HepG2-ADH and HepG2-Vec cells were treated with 50 mM ethanol, and then cellular levels of Brf1 mRNA, tRNA, and 5S rRNA were determined. The results indicate that ethanol markedly enhances the levels of Brf1 mRNA in HepG2-ADH cells, compared to HepG2-Vec cells (Figure 1(a)). Immunoblot analysis indicates that ethanol increases Brf1 expression (Figure 1(b)). We also determined the Brf1 target genes, tRNA^Leu^ and 5S rRNA, to check their alterations after ethanol treatment. The results show that ethanol elevates the transcription levels of tRNA^Leu^ and 5S rRNA in HepG2-ADH cells (Figure 1(c)). To test whether the alteration of Brf1 affects Pol III gene transcription, we transiently transfected HepG2-ADH cells with Brf1 siRNA or mismatch siRNA (MM siRNA) as a control. The results show that decreasing Brf1 expression significantly reduces the cellular levels of tRNA^Leu^ (Figure 1(d)) and 5S rRNA (Figure 1(e)). These studies demonstrate that Brf1 is a key factor of Pol III gene transcription.

### 3.2. How Alcohol Affects Brf1 Expression

**Figure 2:**
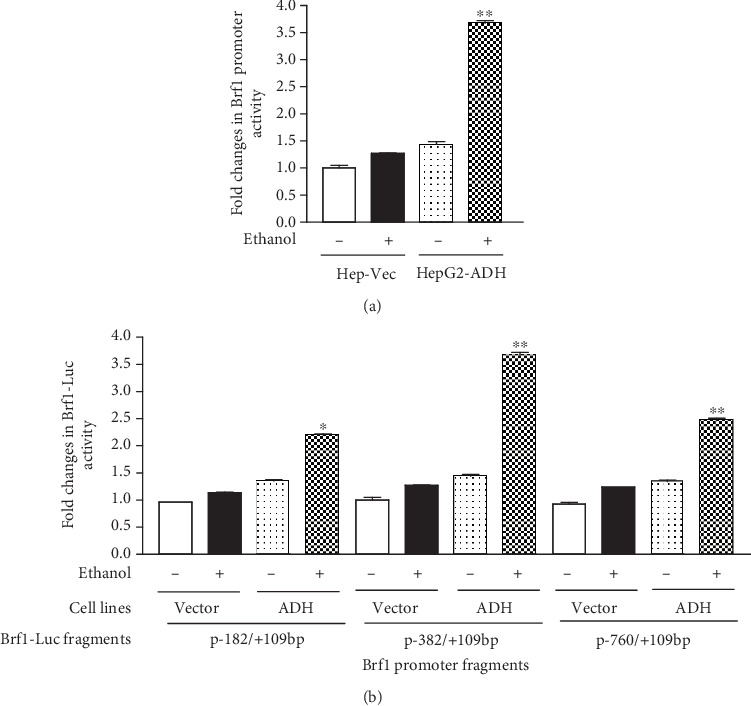
Identification of alcohol-induced response fragments of Brf1 promoter. (a) Alcohol increases Brf1 promoter activity. HepG2-ADH cells were transfected with Brf1-Luc reporter construct (p-382/+109bp) and treated with 50 mM ethanol to determine Luc activity. (b) Identifying alcohol-induced response fragment in Brf1 promoter region. HepG2-ADH and HepG2-Vec cells were transfected with the three different length fragments of Brf1-Luc reporter constructs (p-182/+109bp, p-382/+109bp, and p-760/+109bp) and treated with 50 mM ethanol to determine Luc activity. These results indicate that alcohol-induced high-response region of Brf1 promoter locates at p-382/+109bp of Brf1-Luc construct. The bars represent mean ± SE of at least three independent determinations. ^∗^*p* < 0.05 and ^∗∗^*p* < 0.01.

To explore the mechanism of alcohol-induced Brf1 expression, we generated constructs of human Brf1 promoter luciferase reporter (Brf1-Luc). Both HepG2-ADH and HepG2-Vec cells were transfected with the Brf1 p-382/+109-Luc construct. The result shows that ethanol enhances Brf1 promoter activity in HepG2-ADH cells, compared to control cell line HepG2-Vec (Figure 2(a)). To further identify the alcohol-affected part of the Brf1 promoter, we generated shorter (p-182/+109bp) and longer (p-760/+109bp) constructs and tested their difference of alcohol-induced response of the Brf1 promoter. As we can see, the inductions of Brf1 promoter activities of p-182/+109bp and p-760/+109bp fragments are significantly lower than that of the p-382/+109bp fragment. These results display that the alcohol-caused high-response fragment of the Brf1 promoter locates at the p-382/+109bp region.

### 3.3. Signaling Event of Alcohol-Induced Transcription of Brf1 and Pol III Genes

**Figure 3:**
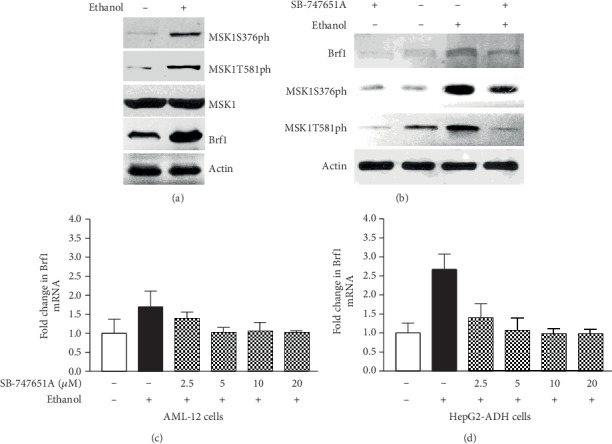
Alcohol induces MSK1 phosphorylation to mediate Brf1 expression. (a) Alcohol induces MSK1 activation. HepG2-ADH cells were starved in FBS-free DMEM for 4 hours and then treated with 50 mM ethanol for another 2 hours. MSK1 phosphorylation at serine 376 and tyrosine 581 of resultant cell lysates was determined with the corresponding antibodies. (b) HepG2 cells were pretreated with MSK1 inhibitor, SB-747651A (10 *μ*M), and treated with 50 mM ethanol. The cellular levels of Brf1 proteins were determined by immunoblot assay against Brf1 antibody. A representative immunoblot is shown in (b). (c, d) AML-12 cells and HepG2-ADH cells were pretreated with SB-747651A, and the cellular levels of Brf1 mRNA were determined by RT-qPCR. The fold changes are calculated by normalizing to the amount of GAPDH mRNA. The bars represent mean ± SE of at least three independent determinations. ^∗^*p* < 0.05 and ^∗∗^*p* < 0.01.

**Figure 4:**
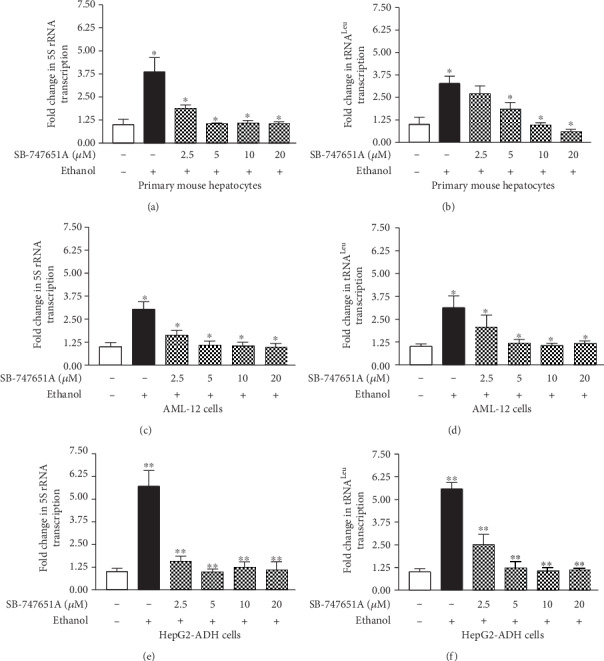
Inhibiting MSK1 pathway decreases alcohol-induced Pol III gene transcription. Primary mouse hepatocytes, AML-12 cells, and HepG2-ADH cells were pretreated with different amounts of SB-747651A and then treated with 50 mM ethanol as described above. Total RNAs were extracted from these cells. Resultant RNAs were used to determine the levels of pre-tRNA^Leu^ and 5S rRNA by RT-qPCR. (a, b) Primary mouse hepatocytes; (c, d) immortalized mouse AML-12 cells; (e, f) engineered HepG2-ADH cells. Left panel: 5S rRNA; right panel: pre-tRNA^Leu^. The fold changes are calculated by normalizing to the amount of GAPDH mRNA. The bars represent mean ± SE of at least three independent determinations. ^∗^*p* < 0.05 and ^∗∗^*p* < 0.01.

**Figure 5:**
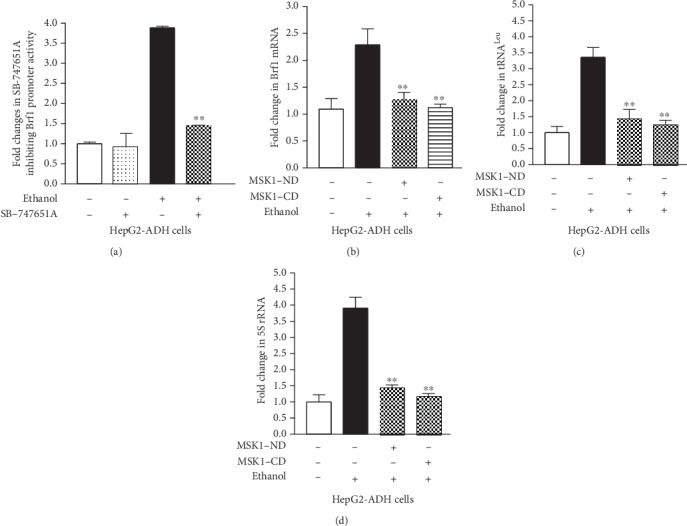
MSK1 mediates Brf1 promoter activity. (a) Inhibiting MSK1 inhibits Brf1 promoter activity. HepG2-ADH cells were transfected with the Brf1-Luc reporter construct (p-382/+109bp), pretreated with 5 *μ*M SB-747651A, and then treated with 50 mM ethanol to determine Luc activity. (b–d) Inactivating MSK1 reduces Brf1 expression and Pol III gene transcription. HepG2-ADH were transfected with inactivated MSK1 at N-terminal or C-terminal expression constructs for 48 hours. The cells were treated with 50 mM ethanol for another 2 hours. Total RNAs were extracted from the cells to determine the levels of Brf1 mRNA (b), pre-tRNA^Leu^ (c), and 5S rRNA (d) by RT-qPCR. The fold changes are calculated by normalizing to the amount of GAPDH mRNA. The bars represent mean ± SE of at least three independent determinations. ^∗∗^*p* < 0.01.

Our early studies have demonstrated that MAP kinases (ERKs, p38, and JNKs) mediate Brf1 and Pol III gene transcription [17, 18]. Further analysis indicates that JNK1 positively, but JNK2 negatively, modulates Brf1 expression [8, 17]. MSK1 is a downstream component of the MAP kinase pathway. Our previous study and others have demonstrated that MSK1 mediates H3ph [26, 27], while H3ph modulates Brf1 expression and Pol III gene transcription [14, 15]. Therefore, we further investigate whether MSK1 modulates the induction of Brf1 and Pol III genes caused by alcohol. The results indicate that alcohol markedly induced MSK1 phosphorylation (MSK1ph), either MSK1ph serine 376 or tyrosine 581 (Figure 3(a)). Inhibiting MSK1 by its specific inhibitor, SB-747651A, decreases the protein level of Brf1 in HepG2-ADH cells (Figure 3(b)), while SB-747651A significantly reduces the levels of Brf1 mRNA in the HepG2-ADH cell (Figure 3(d)) much more than in the AML-12 cell (Figure 3(c)). Next, we have determined changes in alcohol-induced Pol III gene transcription by the inhibitor. The results show that SB-747651A markedly inhibits the levels of tRNA^Leu^ and 5S rRNA transcription in primary mouse hepatocytes (PMH) (Figures 4(a) and 4(b)) and immortalized mouse hepatocytes, AML-12 cells (Figures 4(c) and 4(d)), but dramatically decreases the levels of tRNA^Leu^ and 5S rRNA in HepG2-ADH cells (Figures 4(e) and 4(f)). These results clearly indicate that high doses of SB-747651A display stronger effects of inhibition on Brf1 and Pol III genes (Figures 3(c), 3(d), and 4). As alcohol increases Brf1 promoter activity (Figure 2), we further determine whether MSK1 mediates the function of the Brf1 promoter. HepG2-ADH cells were transfected with Brf1-Luc reporters (p-382/+109bp) and pretreated with SB-747651A. The result indicates that inhibiting the MSK1 pathway dramatically decreases the activity of the Brf1 promoter fragment ( p-382/+109bp) (Figure 5(a)). Therefore, we further investigate how MSK1 affects Brf1 expression. Interestingly, mutated C-terminal or N-terminal domains of MSK1 dramatically repress alcohol-induced Brf1 expression (Figure 5(b)). More interestingly, depriving the two domains also decreases the levels of alcohol-induced tRNA^Leu^ (Figure 5(c)) and 5S rRNA (Figure 5(d)) transcription. Together, these studies demonstrate that MSK1 indeed mediates Brf1 expression and Pol III gene transcription.

### 3.4. Inhibiting MSK1 Pathway Causes Cellular Phenotypic Alteration

**Figure 6:**
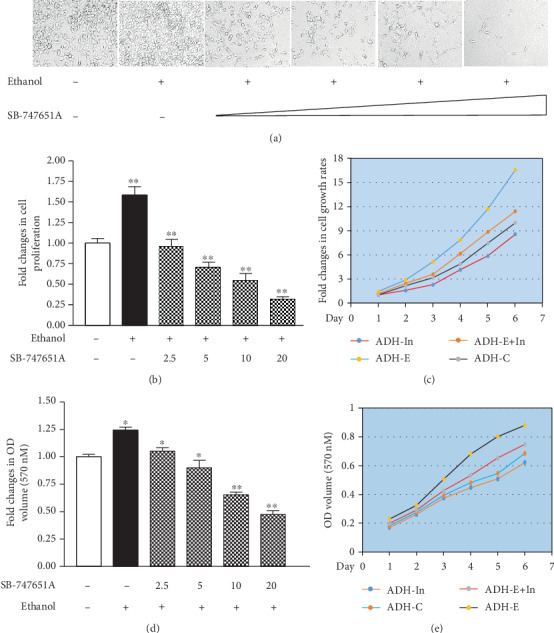
Inhibiting MSK1 pathway caused cellular phenotypic alteration. (a) HepG2-ADH cells were seeded into 6-well plats and grown with different amounts of SB-747651A and 50 mM ethanol. The concentrations of SB-747651A were 0, 2.5, 5, 10, and 20 *μ*M in turn. The pictures were taken under a microscope (Nikon Eclipse TE300). Original magnification ×100. (b, c) The viability and total cell numbers were counted after plated cells. The cells were treated serially by SB-747651A as a dose curve (b) and 50 mM ethanol as indicated. The cells were treated with 10 *μ*M SB-747651A and 50 mM for 1-6 days as a time curve (c). (d, e) The cells were treated as above, and the rates of cell growth were detected by MTT assay. The bars represent mean ± SE of at least three independent determinations. ^∗^*p* < 0.05 and ^∗∗^*p* < 0.01.

**Figure 7:**
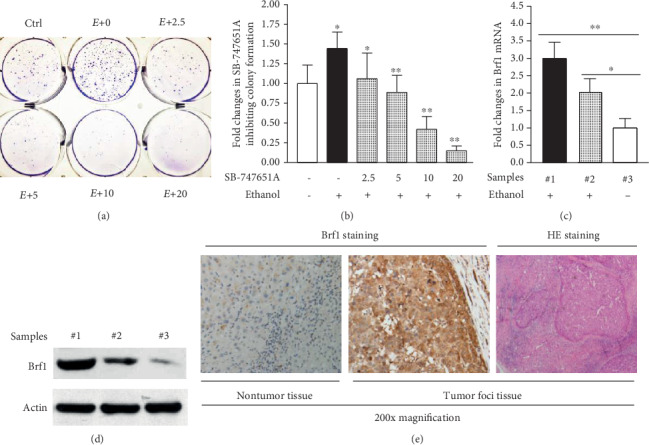
Brf1 expression was increased in tumor tissues. (a, b) Colony formation. HepG2-ADH cells were seed into 6-well plates and treated with SB-747651A and ethanol as described above. The results indicate that MSK1 inhibitor reduces the rates of colony formation. (c, d) Overexpression of Brf1 in tumor foci of mouse liver. Total RNAs and lysates were extracted from the tissues of normal liver (sample #3), alcohol-intake liver (sample #2), and HCC liver with alcohol consumption (sample #1). The levels of Brf1 mRNA were determined by RT-qPCR (c). The protein levels of Brf1 in the tissues were analyzed by immunoblot (d). (e) Brf1 staining. Immunohistochemistry (IHC) staining of Brf1 in paracarcinoma tissues (e, left) and tumor foci (e, middle) of human HCC. H&E staining of HCC tumor tissue (e, right). A representative Brf1 staining of human HCC samples. Magnification ×200. These results clearly show that Brf1 expression was increased in tumor tissues of human HCC.

To determine whether inhibiting the MSK1 pathway affects cellular phenotypes, we performed the assays of cell growth and colony formation of HepG2-ADH cells. The results indicate that ethanol increases the rate of cell proliferation, compared to the control cells without ethanol treatment (Figures 6(a)–6(c)), while SB-747651A dramatically inhibits the induction of cell growth (Figures 6(a)–6(c)). The reductions of the rates of cell growth in high doses of SB-747651A are significantly more than those in lower doses (Figure 6(a)). The effects of the MSK1 inhibitor on cell growth are in dose- and time-dependent manners (Figures 6(b)–6(e)). In addition, we further determined the role of MSK1 in colony formation. An anchor-independent growth assay of HepG2-ADH cells was performed. The results indicate that ethanol increases the rate of colony formation (Figures 7(a) and 7(b)). In contrast, the MSK1 inhibitor significantly represses colony formation of HepG2-ADH cells (Figures 7(a) and 7(b)). These results indicate that inhibiting the MSK1 pathway really causes cellular phenotypic changes.

### 3.5. Brf1 Overexpression in Hepatocellular Carcinoma

The above studies have demonstrated that Brf1 is a key factor for Pol III gene transcription and cell growth. Alcohol enhances Brf1 expression. In contrast, reducing Brf1 expression by inhibiting the MSK1 pathway represses cell growth and colony formation. Therefore, we further investigate Brf1 expression in the liver tumor tissues of mice and humans. Alcohol-feeding HCV NS5A transgenic mouse promoted liver tumor development [7, 29]. Our studies indicate that Brf1 expression, either its mRNA (Figure 7(c)) or protein (Figure 7(d)), is significantly increased in the liver tumor tissue of mouse HCC (#1 in Figures 7(c) and 7(d)), compared with nontumor tissues (#2 and #3 in Figures 7(c) and 7(d)). Our early studies have demonstrated that Brf1 was overexpressed in tumor foci of human HCC cases, compared to paracarcinoma tissue of HCC [16]. The immunohistochemistry stain of Brf1 of human HCC samples shows that Brf1 is overexpressed in tumor foci (Figure 7(e), middle), compared to paracarcinoma tissue of human HCC (Figure 7(e), left). Therefore, these studies are consistent in displaying that increasing Brf1 expression is indeed associated with liver tumor development.

## 4. Discussion

**Figure 8:**
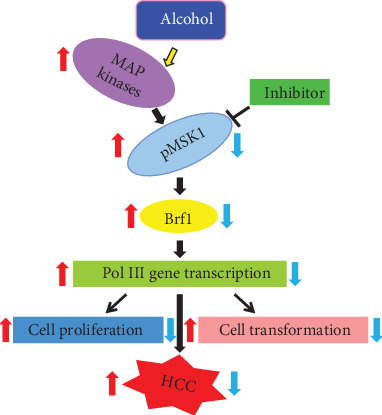
Schematic illustration of MSK1-mediated alcohol-associated HCC. Alcohol activates MSK1 to increase transcription of Brf1 and Pol III genes, resulting in cell proliferation and colony formation, eventually causing HCC. Inhibited MSK1 decreases Brf1 expression to reduce the rates of cancer cell growth and colony formation.↑: increase; ↓: decrease.

In the present study, our results indicate that alcohol enhances transcription of Brf1 and Pol III genes (tRNA^Leu^ and 5S rRNA) in primary mouse hepatocytes, immortalized mouse liver AML-12 cells, and engineered human HCC HepG2-ADH cells. Signaling transduction analysis reveals that alcohol activates the MSK1 pathway to upregulate activities of Brf1 and Pol III genes, while inhibiting MSK1 reduces the alcohol-increased expression of Brf1 and Pol III genes. The inhibitor of MSK1, SB-747651A, reduces the rates of cell proliferation and colony formation. Alcohol feeding promotes liver tumor development of the HCV-NS5A transcription mouse. Further studies show that Brf1 is overexpressed in HCC liver tumor tissues of mice and humans (Figure 8). Mechanism analysis demonstrates that the alcohol-induced highest response region of the Brf1 promoter is at p-382/+109bp. These results, for the first time, show the identification of the alcohol-response promoter site of the key transcription factor, Brf1. These novel outcomes from the study will enhance our understanding of the mechanism of alcohol-associated HCC development.

Studies have demonstrated that upregulation of Brf1 and Pol III genes is associated with cell proliferation, transformation, and tumorigenesis [7, 9–13, 15]. The tRNA and 5S rRNA gene products are increased in both transformed cells and tumor tissues. It implies that they may play an important role in tumor development. Brf1 is a key transcription factor, which is a component of the TFIIIB complex in the transcription machinery of Pol III genes. Brf1 specifically and directly modulates transcription of tRNAs and 5S rRNA. Decrease of Brf1 results in reduction of Pol III gene levels; the reduction is able to repress transformation of cells and xerography formation of the nude mouse [12–15]. Chronic alcohol consumption causes liver fibrosis and cirrhosis and enhances the risk of HCC [33]. Alcohol was classified as carcinogenic by the International Agency for Research on Cancer (IARC) [34–37]. Here, our study indicates that the alcohol-fed HCV-NS5A transgenic mouse increases Brf1 and Pol III gene transcription to facilitate liver tumor development (Figures 7(c) and 7(d)). Brf1 expression is enhanced in tumor tissues of mouse HCC with alcohol feeding, compared to those nontumor liver tissues, such as the liver tissues with or without alcohol intake (Figures 7(c) and 7(d)). Brf1 expression is elevated in the human tumor tissue of HCC (Figure 7(e)). Our study shows that ethanol enhances Brf1 expression and Pol III gene transcription in nontumor hepatocytes, such as PMHs and AML-12 cells, and the tumor liver cell, HepG2-ADH cells (Figure 1), while the induction of Brf1 and Pol III genes in tumor hepatocytes-HepG2-ADH cells is much higher than that in nontumor hepatocytes, PMHs and AML-12 cells. It suggests that upregulation of Brf1 and Pol III genes is closely related to liver tumorigenesis. These studies are consistent with recent reports that Brf1 is overexpressed in breast cancer, gastric cancer, and prostate carcinoma [10, 38, 39].

MAP kinases are an important family of protein kinases, which modulate gene expression, resulting in cell transformation and tumorigenesis. Our early studies have demonstrated that MAP kinases modulate transcription of Brf1 and Pol III genes [7, 8, 17, 18]. MSK1 is a downstream component of MAP kinases, which locates in the nucleus, called the nuclear protein kinase. MSK1 mediates gene expression and cell transformation [19–23]. MSK knockout mice are not significant problems of health [24, 25]. Knockout MSK represses skin cancer development in mice [24]. Our early study has shown that MSK1 mediates H3ph [26], whereas H3ph modulates Brf1 expression as well as transcription of Pol III genes [14, 15]. Inhibiting H3ph decreases the cellular levels of Brf1 and Pol III genes, leading to repression of cell proliferation and transformation [14, 15]. These studies suggest that inhibiting Brf1 expression and Pol III gene transcription may suppress HCC development. Therefore, we explore the role of MSK1 in alcohol-caused upregulation of Brf1 and Pol III genes. Here, our results indicate that alcohol activates MSK1 by inducing MSK1 phosphorylation at its serine 367 and tyrosine 581 (Figure 3(a)). Interestingly, the MSK1 inhibitor, SB-747651A, dramatically inhibits activities of Brf1 and Pol III genes induced by alcohol (Figures 3 and 4). Further analysis indicates that mutant MSK1 in either the N-terminal or the C-terminal depletes alcohol-increased Brf1 and Pol III gene transcription (Figures 5(b)–5(d)). These studies demonstrate that MSK1 indeed mediates the activities of Brf1 and Pol III genes.

Brf1 is a key factor which involves organ development [40]. Although our early studies have indicated that alcohol increases expression of Brf1 and Pol III genes of hepatocytes [7, 33], it remains to be elucidated how alcohol affects Brf1 transcription. To deeply explore the mechanism of alcohol-affected Brf1 expression, we established the constructs of Brf1 promoter-luciferase reports of different lengths, such as p-760/+109bp, p-382/+109bp, and p-182/+109bp. The results reveal that alcohol induces Brf1 promoter activity of the three fragments, while the highest peak of the induction of the promoter activity is at the p-382/+109bp fragment (Figure 2). This indicates that the p-382/+109bp fragment is more sensitive to alcohol treatment than the other two fragments. Namely, it suggests that the alcohol-induced response site of the Brf1 promoter is at this region, p-382/+109bp. Interestingly, the MSK1 inhibitor, SB-747651A, significantly reduces the induction of the Brf1 promoter activity at this site (Figure 5(a)). This implies that the region of p-382/+109bp at the Brf1 promoter is a key response site to alcohol, which is mainly to control alcohol-induced Brf1 transcription.

Moreover, our results indicate that inhibiting MSK1 by SB-747651A dramatically decreases the rates of alcohol-increased cell growth and colony formation (Figures 6, 7(a), and 7(b)), as Brf1 is overexpressed in HCC tumor tissues of animals and humans (Figures 7(c)–7(e)). Our studies have revealed that high Brf1 expression of human HCC cases reveals shorter survival time [16], whereas inhibiting Brf1 expression represses cell transformation and xerography formation of the mouse [12–15]. Here, our results indicate that SB-747651A significantly inhibits alcohol-induced cell growth and colony formation (Figures 6, 7(a), and 7(b)). More interestingly, our recent study indicates that the signals of MSK1S376ph in tumor tissue have been detected by immunofluorescence staining, while the inhibitor SB-747651A significantly repressed tumor growth of nude mice (unpublished data). This implies that inhibiting the MSK1 pathway may be a potential approach for HCC therapy. SB-747651A is a small molecule and a soluble, stable, and absorbable chemical, which specifically and dramatically inhibits MSK1 activity [41]. Therefore, SB-747651A is a potential pharmaceutical agent. More works *in vivo* need to be performed for developing a new therapeutic medicine for HCC using SB-747651A.

## 5. Conclusion

Our studies show that alcohol increases expression of Brf1 and Pol III genes in liver cells. Brf1 expression is elevated in HCC tumor tissues of animals and humans. Alcohol induces activation of MSK1. Inhibiting MSK1 decreases Brf1 and Pol III gene expression, leading to repression of cell growth and colony formation by the MSK1 inhibitor, SB-747651A. Our mechanism analysis demonstrates, for the first time, that alcohol enhances Brf1 transcription through the response fragment (p-382/+109bp) of its promoter. SB-747651A markedly inhibits Brf1 promoter activity. These outcomes from this study show that inhibiting the MSK1 pathway may be an effective approach for HCC therapy. Therefore, developing a novel pharmaceutical agent with SB-747651A will benefit the scientific community and the patients of HCC.

## Data Availability

The data used to support the findings of this study are available from the corresponding authors upon request.

## Abbreviations

HCC: Hepatocellular carcinoma
MSK1: Mitogen- and stress-activated protein kinase 1
ADH: Alcohol dehydrogenase
CYP2E1: Cytochrome P450 2E1
ROS: Reactive oxygen species
ALD: Alcoholic liver disease
TFIIIB: Transcription factor III B complex
Pol III genes: RNA polymerase III-dependent genes
Brf1: TFIIB-related factor 1
PMHs: Primary mouse hepatocytes
NS5A: HCV nonstructural 5A protein
IARC: International Agency for Research on Cancer
MM siRNA: Mismatch siRNA
SiRNA: Small interfering RNA
RT-qPCR: Real-time quantity PCR
H3ph: Histone H3 phosphorylation
Brf1-Luc: Brf1 promoter-luciferase reporter.
